# The Effectiveness of Transabdominal Ultrasonography in Managing Chronic Constipation in the Elderly, with a Focus on the Underlying Pathological Conditions

**DOI:** 10.3390/diagnostics15040476

**Published:** 2025-02-16

**Authors:** Noriaki Manabe, Minoru Fujita, Ken Haruma

**Affiliations:** 1 Division of Endoscopy and Ultrasonography, Department of Clinical Pathology and Laboratory Medicine, Kawasaki Medical School, Okayama 700-8505, Japan; 2 Department of General Internal Medicine 2, Kawasaki Medical School, Okayama 700-8505, Japan

**Keywords:** chronic constipation, elderly people, transabdominal ultrasonography

## Abstract

Chronic constipation is more common in the elderly and associated with numerous diseases. For the diagnosis of chronic constipation in the elderly, it is essential to exclude constipation secondary to colorectal cancer or other causes. Chronic constipation in the elderly also often requires lifestyle modifications, as well as drug treatments because of the involvement of pathologies such as reduced colonic transport capacity and rectal hyposensitivity. Therefore, it is extremely important to evaluate the pathophysiology of both the colon and the rectum. Transabdominal ultrasonography (TUS) is a key technique for providing comprehensive medical care and allows simultaneous functional assessment and exclusion of organic diseases related to constipation such as colorectal cancer. Although several studies have reported the clinical utility of TUS for chronic constipation, which includes its simplicity, noninvasiveness, and low cost, the majority were in children. Thus, there are limited studies in adults. Herein, we review the utility of TUS for indirect assessment of colonic transit time using several TUS parameters that can be applied clinically, as well as treatment options for chronic constipation. The constipation index (i.e., mean transverse diameter of the colon), assessed by TUS, is a useful indirect indicator of colonic transit time. If the constipation index is <21.2, increased fiber or treatment with osmotic agents should be used. If the constipation index is ≥21.2, then the ratio of the left to the right lateral colonic diameters should be evaluated. If this value is ≥0.5, a secretagogue or bile acid transporter inhibitor should be administered. It is noteworthy that nursing care is becoming increasingly important in Japan’s super-aging society. A significant proportion of nursing care is provided to patients with chronic constipation, a cohort that is predicted to grow in the future. In these patients, fecal masses often remain in the rectum, which may require an enema or stool extraction. Therefore, it is important to assess both the presence of feces in the rectum and the consistency of the feces. Recently, portable ultrasound (US) devices equipped with artificial intelligence have been developed and used clinically for treatment of patients with chronic constipation in nursing care. Rectal findings using portable US devices can aid in selecting appropriate constipation treatments. Thus, portable US will likely become increasingly important as a next-generation examination device in nursing care. TUS (including portable US) is noninvasive, simple, and repeatable and will become a fundamental modality in the management of chronic constipation.

## 1. Introduction

Gastrointestinal diseases encompass a range of conditions, from organic diseases such as colorectal cancer to functional diseases such as chronic constipation. It is essential to provide comprehensive medical care in these patients [1]. Recent technological advances have broadened the range of examinations that can be performed with transabdominal ultrasonography (TUS), and TUS is now used clinically for diagnosis of a variety of gastrointestinal diseases, which include malignant and benign neoplastic diseases and vascular lesions [2,3,4,5,6]. Additionally, TUS has been used to evaluate the pathophysiology of various functional gastrointestinal diseases such as functional dyspepsia and pharyngeal dysphagia [7,8,9,10,11]. Thus, TUS is a very useful tool in providing comprehensive medical care.

In this review, we describe the role of TUS in the diagnosis of chronic constipation, which has been increasing in recent years with our super-aging society—note that the role of TUS as a diagnostic test is mainly to rule out secondary constipation. Next, we describe the clinical application of TUS from the perspectives of pathophysiology and treatment of chronic constipation. Finally, the potential integration of TUS into the nursing care of patients with chronic constipation, which is anticipated to become a more prevalent concern, will be discussed.

## 2. Importance of Treating Chronic Constipation in the Elderly

**Figure 1:**
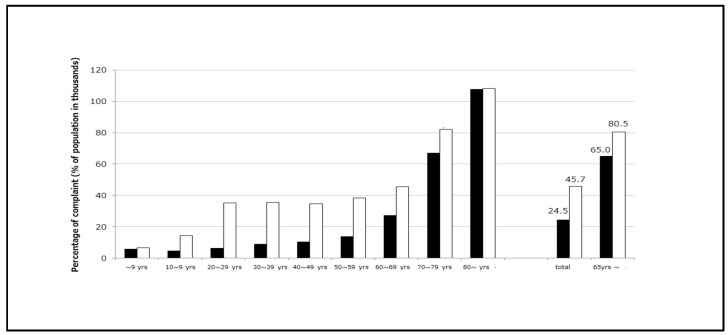
**Prevalence of constipation by gender and age group.** The prevalence of constipation in Japan is approximately 2–5%. Black squares represent men. White squares represent women. Women with constipation are more common in younger age groups, but at ≥70 years (yrs), the male-to-female ratio is almost 1:1. Note: (i) the number of hospitalized patients is not included in the number of people with complaints, but the denominator (the number of household members) includes hospitalized people; (ii) the ‘total number’ includes people of unknown age; (iii) this excludes Kumamoto prefecture. From [12] modified.

The prevalence of constipation by gender and age group is shown in Figure 1 [12]. Given the increasing elderly population in Japan, the number of patients with chronic constipation will inevitably increase. Although chronic constipation is often considered a colorectal disease, recent studies have shown its occurrence in a wide range of diseases in elderly people, which include frailty, dementia, cardiovascular disease, and chronic kidney disease [13,14,15].

The term ‘frail’ is used to describe a state of health that is somewhere between the healthy state and the state requiring long-term care. As indicated in a recent Japanese report [16], chronic constipation is associated with the pre-frail state (stage preceding frailty). In that study, it was emphasized that appropriate treatment and prevention of chronic constipation may prevent patients from progressing to a state requiring long-term care. Chronic constipation was also proposed as part of a vicious cycle that can induce frailty—for example, chronic constipation can cause nutritional disorders related to decreased appetite and sarcopenia [17]. Because this vicious cycle begins to revolve around constipation, appropriate treatment of chronic constipation is important to reduce disease progression.

As indicated in the Cabinet Office’s ‘White Paper on the Aging Society in Fiscal 2009’ [18], the projected number of dementia patients ≥65 years of age (assuming a consistent prevalence of dementia across age groups) is estimated to reach 6.75 million individuals (prevalence rate 18.5%) in Japan by 2025, which equates to approximately 1 in 5.4 individuals. Thus, dementia is a significant health concern in the context of elderly healthcare in Japan. Interestingly, a recent Japanese study examining the relationship between bowel movements, stool consistency, and risk of dementia found that individuals with more severe constipation symptoms (regardless of gender) were at increased risk of developing dementia [19].

The concept of the gut–kidney–heart axis was recently proposed and is likely important in understanding the relationship between chronic constipation, cardiovascular disease, and chronic kidney disease [20]. Specifically, clinical studies have demonstrated that changes in the intestinal microbiota can result in entry of intestinal-derived uremic toxins such as indoxyl sulfate and trimethylamine-N-oxide, a metabolite of the intestinal microbiota, into the bloodstream due to increased mucosal permeability of the intestinal tract, which leads to elevated blood concentrations and an increased risk of cardiovascular disease [21]. Trimethylamine-N-oxide is also associated with increased mortality rates in patients with chronic kidney disease [22]. Furthermore, trimethylamine-N-oxide was found to directly cause vascular damage and fibrosis in the heart and kidney and is thus considered a key factor in the gut–kidney–heart axis. Given these findings, improvement of the intestinal environment and appropriate treatment of chronic constipation may reduce the progression of chronic kidney disease and myocardial fibrosis [23]. In this context, appropriate bowel movement control is an important consideration. A 15-year follow-up study of the impact of functional gastrointestinal disorders on patient prognosis in 3933 Americans ≥20 years old reported a 10% incidence of irritable bowel syndrome and a 16% incidence of chronic constipation [24]. Furthermore, patients with chronic constipation had a lower survival rate than those without chronic constipation. Conversely, there was no evidence of reduced life expectancy in patients with irritable bowel syndrome, chronic diarrhea, functional dyspepsia, or abdominal pain.

Overall, these findings support the idea that chronic constipation is associated with numerous systemic diseases and can impact life prognosis. Therefore, chronic constipation should be regarded as a symptom of systemic diseases rather than a mere colonic issue. Consequently, a tailored approach to treatment that considers the specific underlying condition is essential.

## 3. Clinical Significance of TUS in the Management of Chronic Constipation in the Elderly Population

In the clinical management of chronic constipation, TUS is used as a screening test to rule out secondary constipation and evaluate pathological function, as detailed below.

### Clinical Significance of TUS as a Screening Test to Rule out Secondary Constipation

**Figure 2:**
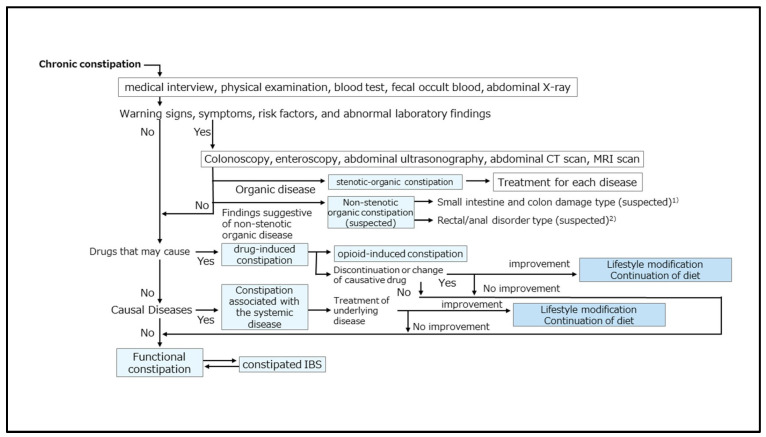
**Flow chart of the diagnosis of chronic constipation.** Note: (1) typical diseases of the small intestine and colon disorders of non-stenotic organic constipation include chronic pseudo-obstruction of the intestine and mega-colon; (2) representative diseases of rectal and anal disorders of non-stenotic organic constipation include rectocele and rectal intussusception. Adapted from the Japanese guidelines for adult patients with chronic constipation (not specific to the elderly). From Ihara et al. [25].

The recently published Japanese guidelines for the treatment of bowel movement abnormalities, as well as the American Gastroenterological Association (AGA)/American College of Gastro-enterology (ACG), describe the diagnostic flowchart for chronic constipation (Figure 2) [25]. This guideline, as well as Western guidelines, clearly states that in patients presenting with chronic constipation, it is critical to first identify any warning signs or symptoms indicative of an underlying organic disease process [26]. These include a sudden change in defecation habits, the presence of bloody stools, unexpected weight loss of >3 kg within a 6-month period, fever, joint pain, and/or abnormal physical findings (e.g., palpation of an abdominal mass, wavelets in the abdomen, palpation of a mass by digital rectal examination, or blood deposits). Given that many patients with chronic constipation are elderly, noninvasive and repeatable TUS may be a valuable tool to rule out constipation secondary to organic or systemic disease when treating chronic constipation.

**Figure 3:**
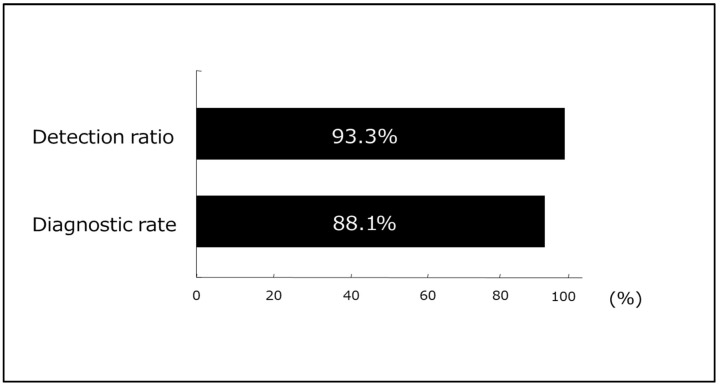
The detection and positive diagnosis rates of various gastrointestinal diseases in 721 patients who presented with abdominal symptoms and underwent prior transabdominal ultrasonography examination.

TUS provides rapid information about the condition of the colon and aids in the selection of appropriate additional testing and treatment. Ultrasound can be used to screen asymptomatic patients to identify several colorectal diseases, which include diverticulosis, inflammatory bowel disease, and cancer. Furthermore, ultrasound is widely available, inexpensive, noninvasive, and does not use ionizing radiation, which makes it safe to use during childhood and pregnancy and means it is repeatable at any time [27]. We previously reported the utility of TUS as a screening test for cases of bloody stool [28] and small bowel lesions [29,30]. The detection and positive diagnosis rates of various gastrointestinal diseases in 721 patients who presented with abdominal symptoms and underwent prior TUS examinations at our institution during the past year are shown in Figure 3. With a 93.3% detection rate and an 88.1% positive diagnosis rate, TUS has sufficient diagnostic ability as a first-line modality and is considered an indispensable screening method, particularly for the elderly.

**Figure 4:**
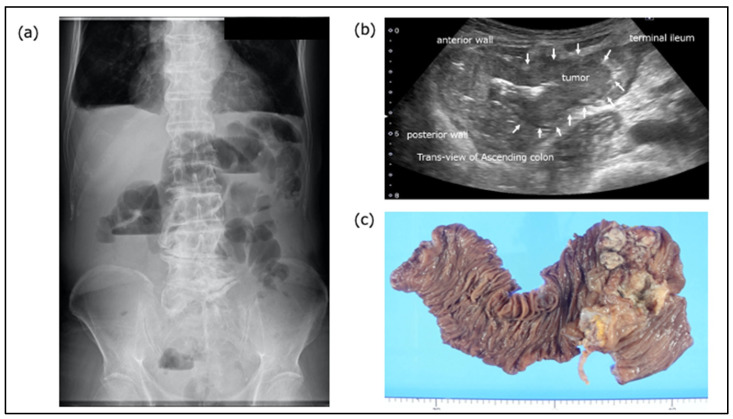
**A case of secondary constipation associated with colon cancer.** Colon cancer is indicated by arrows. (**a**) Abdominal plain X-ray. Air–fluid levels in his dilated small intestine, indicative of a bowel obstruction, are shown. (**b**) TUS image (Aplio i700 TUS-I700; Canon Medical Systems, Otawara, Japan). A dilated terminal ileum is observed, followed by a localized thickened wall lesion with loss of wall stratification in the cecum. (**c**) Resection specimen.

A case of secondary constipation associated with colon cancer is shown in Figure 4. A male patient in his 80s was referred to the gastroenterology outpatient clinic with complaints of abdominal pain and constipation. Abdominal plain X-ray revealed air–fluid levels in his dilated small intestine. In such circumstances, the patient is often reluctant to undergo colonoscopy. TUS (Aplio i700 TUS-I700; Canon Medical Systems, Otawara, Japan) can be performed without any prior preparation. Upon examination, a dilated terminal ileum was observed, as well as a localized thickened wall lesion with loss of wall stratification in the cecum. The patient was diagnosed with an intestinal obstruction caused by cecal cancer.

**Figure 5:**
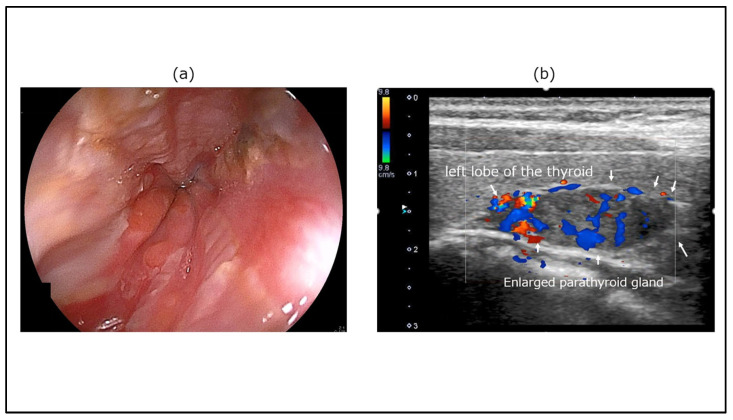
**A case of secondary constipation in the context of endocrine disorders.** (**a**) Upper gastrointestinal endoscopy image. Mucosal damage was observed in the esophagus. (**b**) TUS image (Aplio i700 TUS-I700; Canon Medical Systems, Otawara, Japan). Ultrasonography revealed an enlarged parathyroid mass with abundant blood flow signals in the dorsal thyroid gland. The enlarged parathyroid gland is indicated by arrows.

A patient presenting with secondary constipation in the context of an endocrine disorder is shown in Figure 5. Gastroesophageal reflux disease was found on an upper gastrointestinal endoscopic image of a 50-year-old woman who presented to our clinic with symptoms of upper abdominal pain, anorexia, and constipation. Ultrasonography (Aplio i700 TUS-I700; Canon Medical Systems, Otawara, Japan) revealed an enlarged parathyroid mass with abundant blood flow signals in the dorsal thyroid gland. Various tests indicated that the symptoms were the result of hypercalcemia caused by hyperparathyroidism associated with a parathyroid adenoma.

## 4. Characteristics of Diagnosis and Treatment of Constipation in the Elderly Considering Functional Aspects

The pathogenesis of chronic constipation in the elderly is varied. Lifestyle changes such as decreased physical activity, fluid intake, and food intake can all contribute to the development of constipation [25]. Decreased colonic transport capacity (i.e., colonic transit time) due to aging is also an important pathological abnormality. Additionally, the development of defecation difficulties is often observed in the elderly, which is related to the loss of muscle strength necessary for defecation (e.g., abdominal muscle weakness caused by sarcopenia) [31]. Furthermore, elderly people can show an elevated rectal sensory threshold (i.e., rectal hyposensitivity). Drug treatment is necessary in many of these elderly patients because it is difficult to control their symptoms with lifestyle modification alone [25]. Therefore, for diagnosing chronic constipation in the elderly, it is critical to assess both the colonic transit time and the presence/absence of rectal hyposensitivity, as well as the distribution of stool and gas throughout the colon, particularly in patients with stool and gas accumulation in the rectum.

It is also important to prevent side effects from therapeutic drugs in the elderly to avoid fecal embolization (this is relatively common in elderly patients), a vicious cycle of flailing, and events caused by straining during defecation [31]. The most important consideration is whether the form of the passed stool is normal-to-slightly soft, rather than whether a stool is passed. For example, even if a patient has a daily bowel movement, if the stools are hard, the treatment efficacy will be reduced by half. In elderly patients in particular, sarcopenia and muscle weakness related to defecation, such as in the abdominal muscles, make softer stools desirable. Thus, drug doses should be adjusted with the aim of normalizing stool form rather than only the presence/absence of defecation.

### 4.1. Clinical Trends in TUS for Evaluating the Pathophysiology of Chronic Constipation

A PubMed search for the terms ‘transabdominal ultrasound’ and ‘constipation’ revealed several reports on the clinical application of TUS for chronic constipation (Table 1) [32,33,34,35,36,37,38,39,40,41,42,43]. 

However, most of these studies were in children, in whom functional constipation is a major burden on physical and mental well-being that requires prompt diagnosis. Because of the limitations of both digital rectal examination and abdominal radiography, TUS is considered an alternative technique. An enlarged rectal diameter in children with functional constipation was proposed as a noninvasive diagnostic indicator, and the clinical utility and optimal cutoff value for rectal diameter measured by TUS was reported as a diagnostic criterion for functional constipation in children. For example, Burgers et al. compared transabdominal rectal ultrasonography with digital rectal examination and found a positive correlation in 80% of patients [34]. In that study, using a cutoff value of 30 mm, there was a large overlap between children with functional constipation and healthy controls. As there seems to be a logical relationship between defecation and changes in rectal diameter, patients should be asked about their defecation prior to performing transrectal ultrasound. Because of these limitations, the European Society for Pediatric Gastroenterology Hepatology and Nutrition (ESPGHAN) and North American Society for Pediatric Gastroenterology, Hepatology and Nutrition (NASPGHAN) guidelines state that rectal ultrasound is not routinely recommended for the diagnosis of FC [44].

Recently, the clinical application of TUS for chronic constipation in adults has also been reported. In adults, there is an increasing number of laxative options available, and it has become important to distinguish between them. The American Gastroenterological Association (AGA)/American College of Gastroenterology (ACG), as well as Japanese guidelines, suggest osmotic laxatives as first-line agents and newer laxatives as second-line agents for the treatment of constipation. For this purpose, a simpler, less invasive, and more reproducible method of testing is needed. The clinical application of TUS measurement of the transverse diameter of the colon (as an indirect indicator of colonic transit time) was reported for evaluating the pathophysiology of chronic constipation. Furthermore, portable TUS was used to evaluate defecation control in elderly people with physical and cognitive impairment at long-term care facilities, which is important given the aging society [39,41]. Thus, despite these limited studies, improvements in the utility, convenience, and cost effectiveness of TUS for assessing chronic constipation in adults is expected in the near future.

### 4.2. Clinical Significance of TUS as a Functional Assessment Test

The pathophysiology of constipation can be easily understood when classified using the colonic transit time—normal-transit constipation (NTC), slow-transit constipation (STC), and outlet delay/outlet obstruction (OD). The proportion of each type of constipation varies depending on the age of the patients and enrollment criteria, although OD is reported to account for 50%, STC for 27%, and NTC, including constipation-type irritable bowel syndrome, for 23% [45].

NTC is a type of constipation in which there is no evidence of delayed colonic transit time or impaired bowel evacuation. NTC is thought to be caused by decreased frequency of bowel movements due to decreased mass volume related to low dietary intake or a low-residue diet. Patients with this type of constipation often complain of abdominal pain, and it is highly possible that many cases overlap with constipation-type irritable bowel syndrome [46]. The delayed transit time of the colon in STC is thought to be mainly caused by a decrease or disappearance of the frequency of high amplitude peristaltic contraction, especially in the postprandial period. This is thought to relate, at least in part, to poor control of the nerves involved in peristalsis in the parasympathetic or enteric nervous system in STC. STC is associated with a decreased number or morphological abnormality of Cajal interneurons, and the pacemaker of colonic motility itself is impaired in STC. A small number of patients with STC have chronic dilatation of the colon and are characterized by a lack of response to neostigmine (a cholinesterase inhibitor). In addition to colonic motility, one-third of patients with STC have small intestinal motility disorders, and there are reports of functional abnormalities throughout the gastrointestinal tract [46].

In OD, the weakening of the abdominal and pelvic floor muscles, dysfunction of the anal sphincter, impaired coordination of the puborectalis muscles, and hyporectal sensation impair the defecation mechanism and cause constipation of the impaired stool evacuation type. Approximately 60% of patients with OD have secondary delayed colonic transit constipation, while the cause of constipation remains unknown. It is possible that OD may be secondary to intractable constipation, and OD may be associated with intractable constipation in the patients encountered in daily practice. One-third of OD patients suffer from a lack of proper defecation training in childhood due to behavioral disorders or problems in parent–child relationships. It is difficult to distinguish OD from other types of constipation by interview alone, so anal pressure tests, rectoanal angle measurement by MRI or other imaging tests, and defecography are generally necessary to differentiate OD from other types of constipation. OD can be partially resolved with rectal stimulant laxatives, although long-term treatments such as biofeedback are also required given its pathophysiology [46]. The effectiveness of biofeedback in treating OD was reported to be 70% [47], and it is expected to become widely used in the future.

It should be noted that NTC, STC, and OD are not completely independent of each other and may overlap. Furthermore, the proportion of STC and OD cases may be higher in elderly patients with chronic constipation. Constipation is classified into these three types based on its pathophysiology, and the appropriate treatment should be selected for each type. Unfortunately, however, current constipation treatments are based on trial and error involving drug responses rather than pathophysiology, although pathophysiology-related treatments for chronic constipation are expected in the future.

**Figure 6:**
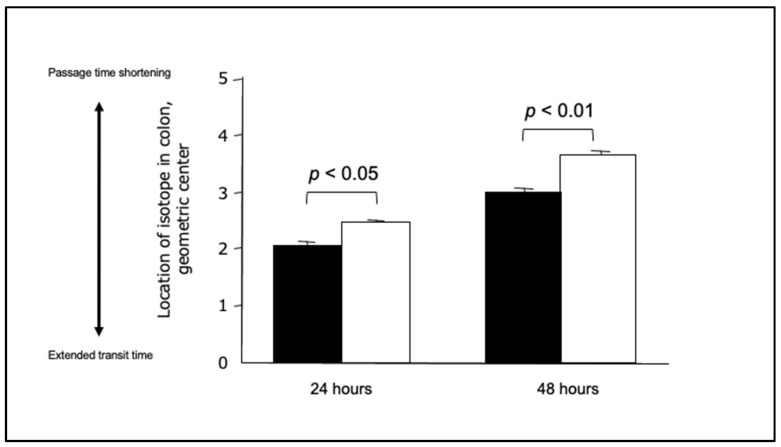
**The difference in colonic transit time between patients with chronic constipation and healthy subjects.** Black squares indicate patients with constipated irritable bowel syndrome/functional constipation. White squares indicate healthy people. The colonic transit time is delayed in chronic constipation patients at both 24 and 48 h. From Manabe et al. [53] modified.

As described, the pathophysiology of chronic constipation is complex and multifaceted [47], with ‘abnormal colonic motility’ emerging as a particularly important aspect [25]. The prevalence of constipation increases with age. Additionally, a reduction in the number of normal ganglion cells in the colonic muscularis was reported with age [48], particularly for Hu-antibody-positive and choline-acetyltransferase-positive cells, but with relative preservation of neuronal nitric oxide synthase neurons [49]. The threshold for rectal perception by balloon extension was also found to increase with age [50,51,52]. It is generally accepted that elderly patients are more susceptible to constipation. Of the various assessments of colonic function, colonic transport capacity (colonic transit time) is regarded as particularly important, as it provides a comprehensive evaluation of colonic function [53]. There are four principal elements essential for the transportation of colonic contents (which are primarily stools)—colonic contractility, harmony of movement of the various parts of the colon, the ability to grasp the stool, and normal colorectal perception. Because the combined action of these factors ultimately moves the stool to the anus, the transit time of the colon is markedly affected by these individual factors alone or in combination. Thus, for evaluating colonic function, colonic transport capacity (colonic transit time) can provide a comprehensive evaluation of these relevant factors [53]. The differences in the colonic transit time between patients with chronic constipation and healthy subjects are shown in Figure 6, which indicates a delay in the colonic transit time at both 24 and 48 h in chronic constipation patients [53].

## 5. Clinical Application of TUS for Evaluating the Colonic Transit Time

**Figure 7:**
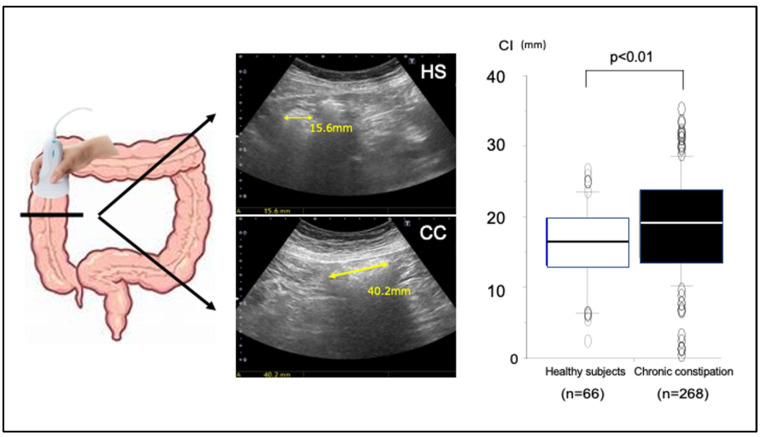
Comparison of the constipation index (CI) between 66 healthy subjects and 268 patients with chronic constipation. CI values for patients with chronic constipation were significantly higher than those for healthy subjects. From Manabe et al. [41] modified.

Despite the development of scintigraphy and radiopaque marker methods for functional assessment of the colonic transit time, the challenges posed by equipment costs, examination duration, and X-ray exposure have impeded their integration into routine clinical practice in Japan. Recently, we reported that TUS can be used for indirect estimation of the colonic transit time through the measurement of colonic lumen diameter [37]. In that study, we initially identified a notable correlation between the colorectal luminal diameter as measured by TUS and computed tomography. Next, using the colorectal luminal diameter obtained by TUS, we derived two essential parameters for assessment of colorectal motility function: (i) the mean transverse colonic diameter (constipation index (CI) = (ascending colon + transverse colon + descending colon + sigmoid colon + rectum)/5) and (ii) the ratio of the luminal diameter of the left-sided and right-sided colon (Left/Right ratio (L/R) = (descending colon + sigmoid colon)/(ascending colon + transverse colon)) [37]. For a long segmental colon such as the ascending colon, measurements were taken at the oral, central, and anal regions and the mean value used. A comparison of these TUS parameters in 66 healthy subjects and 268 chronic constipation patients revealed a significantly higher CI with chronic constipation (Figure 7) [37]. The relationship between the CI obtained by TUS and the colonic transit time obtained by the radiopaque marker method was also examined in 45 eligible patients; there was a significant positive correlation between the two variables, which indicates that patients with a higher CI have longer colonic transit times [37].

## 6. Development of a Treatment Strategy for Chronic Constipation Using TUS

**Figure 8:**
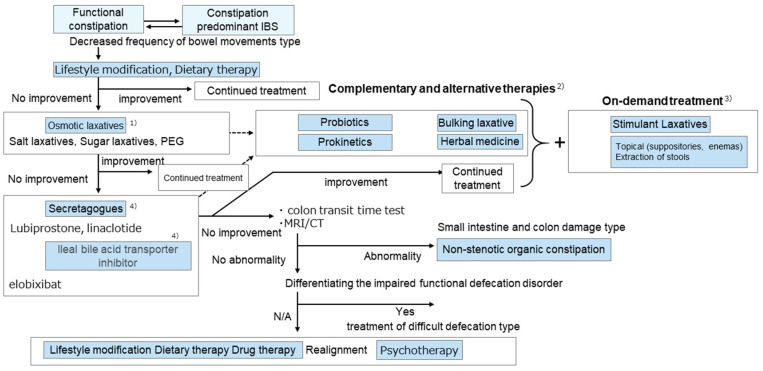
**Flow chart of the treatment of functional constipation.** Note: (1) Magnesium preparations should be used with caution in the elderly and in those with impaired renal function. Serum magnesium levels should be monitored. According to the Pharmaceutical Affairs Law of Japan, lactulose and PEG can be administered to patients with insufficient effects of conventional drug treatment. (2) Patients should be treated according to their condition (e.g., the elderly). Complementary and alternative therapies can be administered in combination with other therapeutic agents. (3) When on-demand therapy is frequently required, a change in the therapeutic agent should be considered. (4) May be administered concomitantly with other therapeutic agents. Adapted from the Japanese guidelines for adult patients with chronic constipation (not specific to the elderly). From Ihara et al. [25].

The treatment flowchart delineated in the ‘Evidence-Based Clinical Guidelines for Chronic Constipation 2023’ is shown in Figure 8 [25]. Several options exist for the treatment of chronic constipation. However, patient responsiveness is based on a trial-and-error approach to drugs. Additionally, the pathophysiology of chronic constipation may result in side effects such as abdominal pain [54]. The ideal treatments for functional diseases should be based on pathophysiology [55,56]. We previously reported a strategy for the treatment of chronic constipation based on pathophysiology derived from TUS data [37]. In that study, 164 patients with chronic constipation were treated with dietary fiber or osmotic laxatives for 2 weeks, and the therapeutic effects were assessed using the CI and L/R parameters [39]. Of these patients, 92 did not respond to the treatment (Group A), while 72 did respond (Group B). Patients in Group A showed significantly higher CI and L/R values. Furthermore, a receiver operating characteristic curve constructed based on these findings showed a cutoff value of 21.2. Thus, although the area under the curve was low, a therapeutic effect for dietary fiber and osmotic laxatives was likely when the CI was <21.2, which is understandable given that CI is an indirect indicator of the colonic transit time. Thus, a high CI value (i.e., in a patient with a prolonged colonic transit time) would indicate that dietary fiber and osmotic laxatives are less effective. In that study, 92 patients who exhibited no response to fiber or osmotic laxatives continued to receive stimulant laxatives for 2 weeks. Of these patients, 50 showed no response to treatment (Group C), while 42 patients demonstrated a favorable response (Group D). Specifically, patients in Group C had a lower L/R ratio than those in Group D, while there were no differences in CI values at this time. Thus, patients with more stools on the right side were less likely to respond to stimulant laxatives.

**Figure 9:**
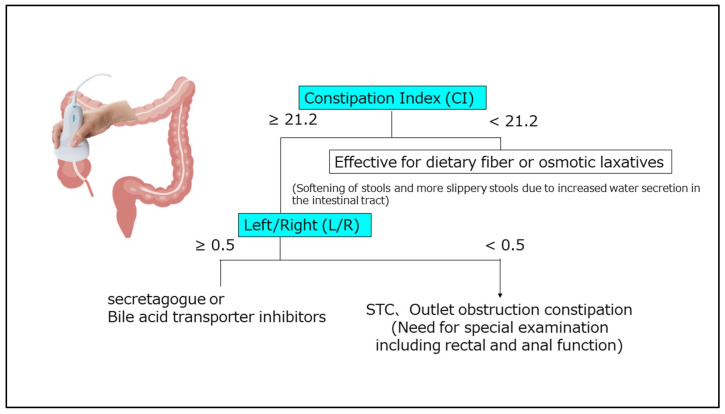
Optimal treatment strategies for patients with chronic constipation. From Manabe et al. [39].

The physiological actions of the colon include admixture, absorption, and fecal transport. Additionally, the functions of the colon can vary depending on the specific site. For example, the ascending and transverse colon areas serve as the primary storage sites for fecal juice, while the descending colon functions primarily as a conduit. The sigmoid colon and rectum are also storage sites, with the latter capable of storing up to 500 mL of fecal matter when transported [57]. In particular, both the transverse and sigmoid colon have fecal storage functions. However, previous studies have demonstrated that the transverse colon has a greater capacity for stretching under the same pressure than the sigmoid colon. Additionally, scintigraphy findings show that isotopes remain in the transverse colon for 24 h, which suggests that this site has the capacity to store stool for an extended period and serves as the primary site for water and electrolyte absorption [57]. This implies that the right-sided colon is more susceptible to stool or gas retention when colonic motility is impaired. Based on these findings, we developed a treatment algorithm for implementation in clinical practice (Figure 9) that uses measurement of the transverse diameter of the colon with TUS [39].

## 7. Toward Development of a Modality for Treatment of Chronic Constipation in Elderly Home-Care Patients

The prevalence of constipation in elderly home-care patients was reported as 56.9%, and it is noteworthy that patients with chronic constipation include those receiving treatment at hospitals and those at home [58]. The need for nursing care is expected to increase in the future, and all medical personnel responsible for community medical care should support initiatives to establish a modality that enables many different professions to participate in the treatment of chronic constipation [59,60]. The diagnostic modalities most frequently recommended for the evaluation of rectal and chronic constipation include plain abdominal radiography, barium enema, colonoscopy, defecography, abdominal computed tomography, and magnetic resonance imaging [61,62,63]. However, these tests have inherent limitations, which include invasiveness, radiation exposure, prolonged examination times, and an inability to provide comprehensive information. Furthermore, these tests are relatively expensive, unsuitable for follow-up examinations, and not standardized. While radiography can be performed in many hospitals, it is moderately expensive and the findings from it are often unclear. By contrast, conventional ultrasonography has the advantages of being rapid, cost effective, noninvasive, safe, and not using ionizing radiation, which makes it widely applicable in clinical practice. Note that all of these tests can only be performed in a hospital setting, which limits their applicability for nursing care.

**Figure 10:**
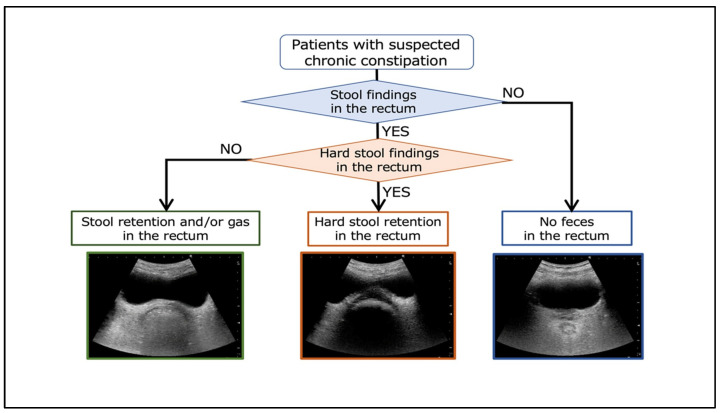
Attempts to develop a method to evaluate pathological function by rectal ultrasound. From Matsumoto et al. [64].

The Chronic Constipation Ultrasound Study Group, which is an affiliated research group of the Diagnosis and Treatment of Chronic Constipation Study Group comprising professionals from various disciplines involved in the treatment of chronic constipation, recently proposed a straightforward functional evaluation method for the treatment of chronic constipation using rectal ultrasound [64]. This method categorizes the state of rectal fecal retention into three patterns and is scheduled for clinical application to aid development of a treatment plan (Figure 10). This examination uses the wireless iViz air^®^ (Fujifilm, Tokyo, Japan) ultrasonography device that can be used in nursing care. This system is equipped with artificial intelligence and can guide the user to the site of the rectum, as well as identify the presence/absence of stools in the rectum. In the absence of fecal matter in the rectum identified using this system, administration of an enema is unnecessary. Furthermore, the identification of hard stools avoids potential complications such as serious colonic perforation that can result from the use of laxatives or other pharmaceutical agents. Moreover, if normal stools are confirmed in the rectum, suppositories can be used to stimulate defecation, facilitating a more targeted and efficacious approach to constipation treatment [65].

The treatment of constipation requires the input of both physicians and a multidisciplinary team that includes co-medical staff. Additionally, given that many patients with chronic constipation are elderly, it is not uncommon for patients to be pre-frail or frail. Thus, the treatment of chronic constipation should be based on the evaluation of pathological function using TUS, including a portable US, which is a noninvasive, simple, and repeatable examination method.

## 8. Conclusions

The recently published Japanese guidelines for the treatment of bowel movement abnormalities describe the clinical application of TUS for adult patients with chronic constipation [25], which differs from that in Western countries. Specifically, clinical question 4-2 of the guidelines poses whether TUS is a useful tool for evaluating the pathophysiology of chronic constipation. The guideline states ‘Although there are few reports of clinical application of TUS in the evaluation of pathophysiology of chronic constipation, it may be useful, and they suggest that it be performed.’ While there is currently a paucity of evidence and the recommendation is therefore weak [33,39,43], there are high expectations for future developments. The treatment of chronic constipation requires the input of physicians and staff from a range of other professions, including non-medical staff. Furthermore, many patients with chronic constipation are elderly and often in a state of frailty. In many advanced countries, TUS is rarely used to evaluate the pathophysiology of chronic constipation. Considering that TUS is a noninvasive, low-cost, and highly repeatable examination method, its utility is recognized by physicians and in the nursing field in Japan. Thus, the clinical application of TUS is increasing, as are reports on its utility in patients with chronic constipation. We envisage that TUS will soon form the basis of the management of chronic constipation in both hospital and nursing care patients.

## Figures and Tables

**Table 1:** Clinical trends in TUS for the evaluation of the pathophysiology of chronic constipation.

Year	Journal	Authors	Subjects	Disease	Methods	Results
2007	Pediatr Radiol.	Bijoś A, et al. [32]	children	functional constipation	transverse diameter of the rectal ampulla by TUS	TUS assessment of stool retention and overfilling of the colon in children with functional chronic constipation has a high correlation with proctoscopy findings and colonic transit time.
2008	J Urol.	Joensson IM, et al. [33]	children	constipation	transverse rectal diameter measured by TUS	Transverse rectal diameter is useful for screening and treatment efficacy.
2013	J Urol.	Burgers R, et al. [34]	children	constipation	Evaluation of rectal mass by TUS	TUS is a noninvasive and reliable alternative to assess the rectal filling state
2015	J Pediatr Urol.	Mason MD, et al. [35]	children	constipation	transverse diameter of the rectal ampulla by TUS	Rectal evaluation by TUS does not correlate with patient symptoms.
2018	Healthcare (Basel).	Tanaka S, et al. [36]	adult	constipation	transverse diameter of the rectal ampulla by TUS	TUS can be used by nurses to visualize rectal fecal retention as constipation patterns in the older people with physical and cognitive impairment at long-term care facilities.
2018	Int J Colorectal Dis.	Manabe N, et al. [37]	adults	constipation	stool and/or gas distribution by TUS	Stool and/or gas distribution of patients with chronic constipation, which is an indirect indicator for colonic transit time.
2019	Arq Gastroenterol.	Momeni M, et al. [38]	children	constipation	Rectal diameter and rectal wall thickness by TUS	Increased rectal diameter and increased rectal wall thickness are associated with constipation.
2019	JGH Open.	Manabe N, et al. [39]	adults	constipation	stool and/or gas distribution by TUS	Evaluation of stool and/or gas distribution by TUS could help physicians predict favorable outcomes with laxatives without side effects for this patient population.
2021	J Paediatr Child Health.	de Abreu GE, et al. [40]	children and adolescents	functional constipation	Rectal diameter by TUS	There was no association between the current cut-off point characterising the rectum as distended and the severity of urinary symptoms, even when functional constipation was present.
2022	J Paediatr Child Health.	Shapouri S, et al. [41]	children	functional constipation	Rectal diameter and rectal wall thickness by TUS	Rectal transverse diameter and rectal anterior wall thickness increased with increasing constipation duration and the presence of hard stools and decreased with increasing frequency of defaecation.
2023	J Paediatr Child Health.	Gatzinsky C, et al. [42]	infants	healthy subjects and functional constipation	Rectal diameter by TUS	Infants diagnosed with functional constipation did not have a greater transverse rectal diameter than infants without, either before or after treatment.
2024	J Bodyw Mov Ther.	Ashrafi A, et al. [43]	adults	functional constipation	pelvic floor muscles by TUS	There is no significant association between the bladder base displacement and the development of functional constipation.

## Data Availability

No new data were created or analyzed in this study. Data sharing is not applicable to this article.

## Abbreviations

The following abbreviations are used in this manuscript: Constipation index (CI); high amplitude peristaltic contraction (HAPC); left/right ratio (L/R); neuronal nitric oxide synthase (nNOS); normal-transit constipation (NCT); outlet delay/outlet obstruction (OD); slow-transit constipation (STC); transabdominal ultrasonography (TUS).
